# A prospective clinical and biological database for pancreatic adenocarcinoma: the BACAP cohort

**DOI:** 10.1186/s12885-018-4906-4

**Published:** 2018-10-16

**Authors:** Cindy Canivet, Sophie Gourgou-Bourgade, Bertrand Napoléon, Laurent Palazzo, Nicolas Flori, Pierre Guibert, Guillaume Piessen, Dominique Farges-Bancel, Jean-François Seitz, Eric Assenat, Véronique Vendrely, Stéphanie Truant, Geoffroy Vanbiervliet, Philippe Berthelémy, Stéphane Garcia, Anne Gomez-Brouchet, Louis Buscail, Barbara Bournet, The BACAP Consortium, Barbara Bournet, Cindy Canivet, Louis Buscail, Nicolas Carrère, Fabrice Muscari, Bertrand Suc, Rosine Guimbaud, Corinne Couteau, Marion Deslandres, Pascale Rivera, Anne-Pascale Laurenty, Nadim Fares, Karl Barange, Janick Selves, Anne Gomez-Brouchet, Bertrand Napoléon, Bertrand Pujol, Fabien Fumex, Jérôme Desrame, Christine Lefort, Vincent Lepilliez, Rodica Gincul, Pascal Artru, Léa Clavel, Anne-Isabelle Lemaistre, Laurent Palazzo, Jérôme Cros, Sarah Tubiana, Nicolas Flori, Pierre Senesse, Pierre-Emmanuel Colombo, Emmanuelle Samail-Scalzi, Fabienne Portales, Sophie Gourgou, Claire Honfo-Ga, Carine Plassot, Julien Fraisse, Frédéric Bibeau, Marc Ychou, Pierre Guibert, Christelle de la Fouchardière, Matthieu Sarabi, Patrice Peyrat, Séverine Tabone-Eglinger, Caroline Renard, Guillaume Piessen, Stéphanie Truant, Alain Saudemont, Guillaume Millet, Florence Renaud, Emmanuelle Leteurtre, Patrick Gele, Eric Assenat, Jean-Michel Fabre, François-Régis Souche, Marie Dupuy, Anne-Marie Gorce-Dupuy, Jeanne Ramos, Jean-François Seitz, Jean Hardwigsen, Emmanuelle Norguet-Monnereau, Philippe Grandval, Muriel Duluc, Dominique Figarella-Branger, Véronique Vendrely, Clément Subtil, Eric Terrebonne, Jean-Frédéric Blanc, Etienne Buscail, Jean-Philippe Merlio, Dominique Farges-Bancel, Jean-Marc Gornet, Daniela Geromin, Geoffroy Vanbiervliet, Anne-Claire Frin, Delphine Ouvrier, Marie-Christine Saint-Paul, Philippe Berthelémy, Chelbabi Fouad, Stéphane Garcia, Nathalie Lesavre, Mohamed Gasmi, Marc Barthet, Vanessa Cottet, Cyrille Delpierre

**Affiliations:** 1 The Department of Gastroenterology and Pancreatology, CHU - Rangueil and the University of Toulouse, 1 avenue Jean Poulhès, TSA 50032, 31059 Toulouse Cedex 9, France; 2 The Biometrics Unit – CTD Inca, the Cancer Institute and the University of Montpellier, Montpellier, France; 3 The Department of Gastroenterology, Jean Mermoz Hospital, Ramsay Générale de Santé, Lyon, France; 4 The Department of Endoscopy, Trocadéro Clinic, Paris, France; 5 The Department of Oncology, the Cancer Institute and the University of Montpellier, Montpellier, France; 6 The Department of Oncology, Leon Bérard Institute, Lyon, France; 7 Univ. Lille, Department of Digestive and Oncological Surgery, Claude Huriez University Hospital, F-59000 Lille, France; 8 The Department of Internal Medicine, Saint-Louis Hospital and Paris 7 Diderot University, Paris, France; 9 The Department of Oncology, CHU - La Timone and the University of Marseille, Marseille, France; 10 The Department of Oncology, CHU - ST-Eloi and the University of Montpellier, Montpellier, France; 11 The Department of Oncology and Radiotherapy, CHU - Haut-Levêque and the University of Bordeaux, Bordeaux, France; 12 The Department of Digestive Surgery and Transplantation, the CHU and the University of Lille, Lille, France; 13 The Department of Gastroenterology, CHU - L’Archet and the University of Nice, Nice, France; 14 The Department of Gastroenterology, Pau Hospital, Pau, France; 15 The Department of Pathology, CHU - Nord and the University of Marseille, Marseille, France; 16 The Biobank, the Cancer Institute and the University of Toulouse, Toulouse, France

**Keywords:** Pancreatic adenocarcinoma, Biobank, Cohort study, Biomarkers, Outcomes, DNA, RNA, Quality control

## Abstract

**Background:**

The prognosis for pancreatic cancer remains poor despite diagnostic advances and treatments with new chemotherapeutic regimens. The five year survival rate remains below 3%. Consequently, there is an urgent need for new treatments to significantly improve the prognosis. In addition, there is a big gap in terms of the screening, early diagnosis and prevention of pancreatic cancer the incidence of which is increasing dramatically.

**Methods:**

Design: the BACAP cohort is a prospective multicenter pancreatic cancer cohort (pancreatic ductal carcinoma) with clinical and multiple biological samples; Participating centers: 15 French academic and private hospitals; Study Population: any cytologically and/or histologically proven pancreatic carcinoma regardless of the stage (resectable, borderline, locally advanced or metastatic) or treatment (surgery, palliative chemotherapy, best supportive care). At least 1500 patients will be included. Clinical data collected include: disease presentation, epidemiological and social factors, baseline biology, radiology, endoscopic ultrasound, staging, pathology, treatments, follow-up (including biological and radiological), and survival. All these data are collected and stored through an e-observation system at a centralized data center. Biological samples and derived products (i.e. before any treatment): blood, saliva, endoscopic ultrasound-guided fine needle aspiration materials from the primary tumor, fine needle biopsy of metastases and surgically resected tissue. DNA and RNA are extracted from fine needle aspiration materials and are quantified and characterized for quality. Whole blood, plasma and serum are isolated from blood samples. Frozen tissues were specifically allocated to the cohort. All derived products and saliva are stored at − 80 °C. Main end-points: i) to centralize clinical data together with multiple biological samples that are harmonized in terms of sampling, the post sampling process and storage; ii) to identify new molecular markers for the diagnosis, prognosis and possibly the predictive response to pancreatic cancer surgery and or chemotherapy.

**Discussion:**

The BACAP cohort is a unique prospective biological clinical database that provides the opportunity to identify correlations between the presence/expression of a broad panel of biomarkers (DNA, RNA, miRNA, proteins, etc.), epidemiological and social data, various clinical situations, various stages and the differentiation of the tumor, treatments and survival.

**Trial registration:**

ClinicalTrials.gov Identifier: NCT02818829. Registration date: June 30, 2016.

## Background

Pancreatic ductal adenocarcinoma (PDAC) is the fifth leading cause of cancer-related death in western countries [1]. A recent North American analysis on death projection for the “top cancer killers” due to demographic changes placed pancreatic cancer as the second cause of death by cancer in 2030 after pulmonary carcinoma [2]. Europe is not unaffected by this bleak forecast because the predictions for 2025 are similar here [3–5]. In France the situation is critical. In terms of the various birth cohorts (i.e. from 1920 to 1950), the probability of developing a pancreatic cancer before age 75 has increased [4]. The standard rate of mortality has of course increased in parallel, especially in women [4]. These findings are alarming; a further increase in the incidence of pancreatic cancer is expected over the next 15 years in France regardless of age or gender. We do not have a clear explanation of why this is so. The proportion that is attributable to risk factors can be estimated at 70%, excluding aging (10 to 15%) and the increasing population size (15 to 20%). Genetic factors account for only 10% of the cases (Peutz Jeghers syndrome, FAMM syndrome, Lynch syndrome, hereditary pancreatitis, etc.) [1, 5]. The following risk factors were established for the remaining so-called “sporadic” cases: chronic calcific pancreatitis, tobacco, diabetes, obesity, alcohol use, a high-calorie diet, heavy metals and toxins such as pesticides, benzene, lead and arsenic (with a low relative risk ranging from 1.21 to 2) [5–9].

The prognosis for PDAC remains poor despite diagnostic advancements whereby high resolution imaging such as endoscopic ultrasound (EUS) with fine needle aspiration biopsy (FNAB) [10, 11] and treatments with new chemotherapeutic regimens (gemcitabine, FOLFIRINOX and nab-paclitaxel) [12–14] are used. In fact, a vast majority (85%) of patients are diagnosed with locally advanced tumors and/or metastases due to the lack of specific symptoms and early markers for this otherwise highly aggressive disease. Substantial efforts have been made in basic and clinical PDAC research over the past 20 years, including wide-scale molecular analysis, an improvement in the selection of patients for surgery (differentiating ‘borderline’ from non-resectable tumors), in surgical resection with special attention to ‘tumor resection margins’, new chemotherapy regimens, and a better approach to best supportive care [1]. On the whole, palliative and adjuvant chemotherapy have improved clinical status and overall survival [12–14]. However, although the overall survival of patients who receive palliative or curative treatment has doubled in 15 years, the five-year survival rate remains below 3% [1, 2, 4].

Consequently, there is an urgent need for new treatments to significantly improve the prognosis. In addition, there is a big gap in terms of the screening, early diagnosis and prevention of PDAC. In an effort to respond to all these critical issues, a national multicenter program was launched by the French national institute of cancer (Inca) in 2012. The aim of this program was to form national cohorts that include clinical and epidemiological data linked to biological resources. We proposed the creation of a large French national prospective biobank dedicated to PDAC, fully and financially supported by Inca. This biobank includes clinical, epidemiological and social data linked to biological samples such as blood, serum, plasma, saliva, DNA and RNA from tumor cells and biopsies. The final goal is to correlate all these biological analyses with epidemiological, clinical and follow-up data as well as prognosis. In this paper, we provide a report on the design and management of the French national BACAP cohort (BACAP for ‘Base Clinico-Biologique de l’Adénocarcinome Pancréatique’ - Biological Clinical Pancreatic Adenocarcinoma Database).

## Methods/design

### Aims

The general mission of this prospective project is to provide a clinical biological database of patients with pancreatic adenocarcinoma for the scientific community (requirement of the Inca proposal for Biological Clinical Databases 2012).

This national biological clinical database aims to centralize clinical data (including epidemiological data, treatment and prospective follow-up) along with multiple biological samples that are harmonized in terms of sampling; the post sampling process (including nucleic acid extraction) and storage.

Epidemiological, clinical and translational research must be conducted from this biological clinical database in order to improve understanding of the PDAC epidemiology and to define new biomarkers for the diagnosis, prognosis and follow-up of PDAC patients.

In addition to the prospective design, one of the specific features of the BACAP cohort is the inclusion of all types of PDAC patients. This means, not only those with resected tumors, but also those with locally advanced and/or metastatic tumors. It should be noted that patients with locally advanced and/or metastatic tumors correspond to more than 80% of the cases in “real life”. Consequently, comparative studies between the different groups of patients can be conducted in terms of stage, survival, response to treatment, especially for molecular subtyping for DNA, RNA or protein, and for both in terms of tumor and blood levels.

### Cohort design

#### General design

The BACAP is a national prospective cohort including patients with PDAC that has been cytologically or histologically proven through EUS-FNA, radiologically-guided biopsy, surgical biopsy or a resected specimen. This project is coordinated by the University Hospital of Toulouse under the scientific responsibility of Prof Barbara Bournet. The patients are informed of the goal and the design of the project, agree to participate and sign a specific informed consent. All these patients are followed-up until death. They are recruited from 15 French public hospitals or private health institutions. These participating institutions are: Toulouse University Hospital, Jean Mermoz Private Hospital in Lyon, Clinique du Trocadero in Paris, the Léon Bérard Cancer Center in Lyon, the Cancer Institute of Montpellier, Saint-Louis University Hospital in Paris, Northern University Hospital and La Timone University Hospital, both in Marseille, Nice University Hospital, Pau Hospital, Bordeaux University Hospital, Montpellier University Hospital and Lille University Hospital.

#### Governance

**Fig. 1:**
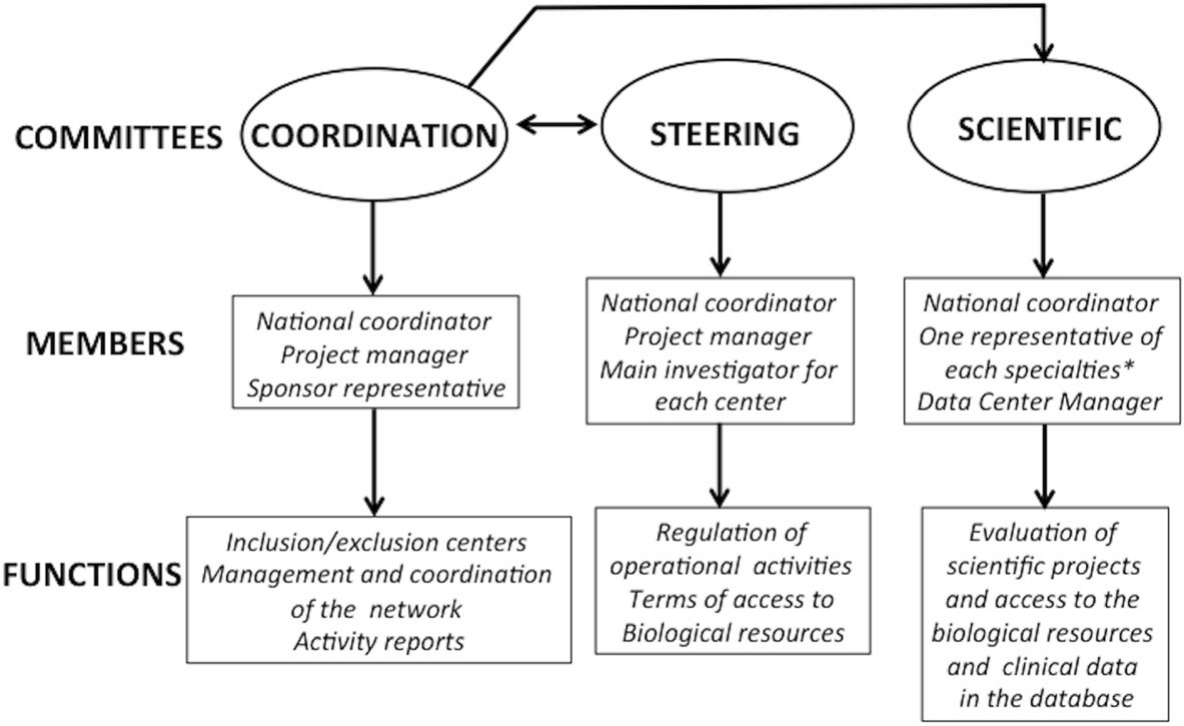
Governance of the BACAP cohort. Details of the various committees responsible for the governance of the cohort with their members and main functions (*: oncology, gastroenterology, pathology, epidemiology, surgery, basic science)

The BACAP was formed by a network of clinicians with the support of two epidemiological research teams and a datacenter. Dedicated governance was first implemented in order to establish this national database. It includes three committees: coordination, steering and scientific. The organization of the BACAP governance including the composition and missions of these committees are detailed in Fig. 1. The coordination committee liaises between the other two. The steering committee validates all decisions made by the scientific committee.

### Legal requirements and ethics

This biological clinical project/database meets all necessary legal requirements and was approved by: i) the national committee for data processing related to health research (Comité Consultatif sur le Traitement de l’Information en matière de Recherche dans le domaine de la Santé, CCTIRS, September 2013, Folder 13.490); ii) the National Data Protection Authority (Commission Nationale de l’Informatique et des Libertés, CNIL, − March 2014, Authorization N°913,462); iii) The ethics committee (Comité de Protection des Personnes pour la recherche biomédicale Sud-ouest et Outre-Mer I, CPP, March 2014). The biological collection was declared to and approved by the French Ministry of Research under the number DC-2013-1974 and the database was declared in Clinical Trials under the number: NCT02818829 (Biological and Clinical Database for Pancreatic Adenocarcinoma).

Whenever the participation of a site was initiated, a meeting was organized with the principal investigator, oncologists, surgeons, gastroenterologists, pathologists and the site clinical research associate dedicated to the study to explain all the procedures of the project and to answer questions.

### Patient population

#### Clinical and biological data collection and follow-up

**Fig. 2:**
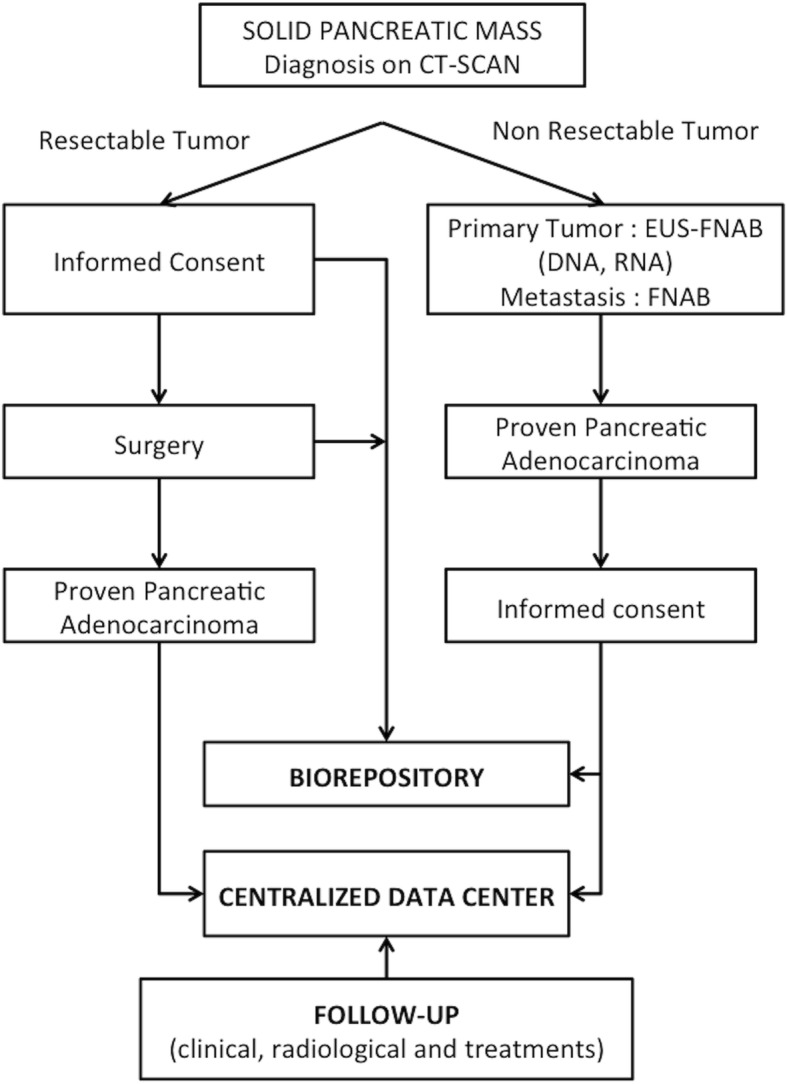
Chart-flow of the BACAP cohort. (EUS-FNAB: Endoscopic ultrasound-guided fine needle aspiration biopsy; FNAB: fine needle aspiration biopsy)

Data are collected at the inclusion of the patient, during the diagnosis process, at the first and possibly subsequent cancer treatment(s) (chemotherapy and/or surgery and/or best supportive care) and through follow-up right up until death. Table 1 details the main socio- demographic, clinical, biological, radiological and histological data that are collected and stored through an e-observation system at a centralized data center (see below). The flow chart of the BACAP study is shown in the Fig. 2 (details of the governance and organization of the cohort are also described at https://www.chu-toulouse.fr/-bacap-project). It should be noted that follow-up and the frequency of follow-up visits depend on the practices of each center.

**Table 1:** Main clinical items collected in the BACAP cohort

Type	Data collected
Socio-demographic	Age, gender, medical history and medication use, work and educational level, alcohol and tobacco consumption, BMI, family history of cancer
Symptoms	First symptoms of the disease, period between first symptoms and first consultation, venous or arterial thrombosis, metastasis (lymph nodes, ascites), performance status with WHO score
Biology	Standard including liver enzymes, lipids, coagulation, CA 19–9
Diagnosis	Radiology, EUS, MRI and PET-scan, pre-therapeutic staging (CT-scan and EUS), cytopathology,
Pathology	Fine needle biopsy or resected specimen: differentiation, lymph nodes, metastases, R status and TNM staging
Treatment(s)	Chemotherapy (first, second and possibly third line), surgery, neo-adjuvant treatment, adjuvant treatment, best supportive care
Follow-up	For each visit, the following were collected: standard laboratory tests, CT scan evaluation, chemotherapy treatment, treatment toxicities, and supportive care. The follow-up ended on the date of death.

### Data management

The database is managed by the Montpellier Cancer Institute Data Center with the Clinsight® software. A Data-management plan and a consistency check program were established when the database was built. Every investigator has a specific usernames and password to connect to the database. A specific and secured login was established to monitor the database. Quality control of the data is done in four ways: i) automatic data consistency check; ii) data management control through regular sending of queries; iii) regular e-control of entered data by the project manager; iv) on-site data monitoring of at least 10% of the data entered. A fill rate analysis plan is also regularly sent to the BACAP coordinator by the data manager.

### Sample collection

Biological samples (blood, saliva, biopsies) are collected at enrollment and before any treatment with chemotherapy and/or radiotherapy, and/or surgical resection. A sample verification process to ensure that the various sites all meet an adequate level of quality is a requirement. Sample tracking was established that includes the time from removal to freezing. Detailed standard Operating Procedures were given to each site. Sample transportation has to be less than 4 h between removal and freezing. A summary of the samples collected in the BACAP cohort is given in Table 2.

**Table 2:** Samples collected in the BACAP cohort

Sample	Post sample treatment	Storage
Blood	Plasma, Total Blood, Serum	- 80 °C
Biopsy (*)	DNA, RNA	- 80 °C
Saliva	–	- 80 °C
Tissue	Frozen	- 80 °C

*: material from EUS-guided fine needle aspiration biopsy of a primary tumor or biopsy from a metastasis

#### Fine-needle aspiration samples

After each Endoscopic ultrasound-guided fine-needle aspiration (EUS-FNA) process, needle flushing is done as previously described [15, 16] in an Eppendorf cryovial containing 500 μl of RNAprotect Cell Reagent® (Qiagen, Courtaboeuf, France) for subsequent DNA and RNA extraction from tumor cells. All subsequent procedures for DNA and RNA extraction and quality control are centralized on the tumor molecular biology platform in Toulouse (the Department of Pathology, the University Institute for Cancer, Toulouse-Oncopole, France). Both DNA and RNA are extracted using the Qiagen Allprep purification Kit® (Qiagen, Courtaboeuf, France). Quantification of both DNA and RNA is subsequently performed using QUBIT dsDNA and RNA BR assay Kits® respectively (ThermoFisher Scientific, Illkirsch, France). Quality control of nucleic acids is then performed using the Agilent 2200 Tape Station Bio Analyzer System® (Agilent Technologies, Santa Cruz, CA, USA). The DNA Integrity Number was calculated (DIN range 1.3 to 9); a value > 6 was a guarantee of DNA quality. The RNA Integrity Number was also calculated (RIN - range 1.8 to 9.9); a value > 7 was a strong guarantee of RNA quality. However, depending of the tissues and the assay, the RIN is not the only critical measurement with which the quality of RNA can be evaluated and the success of subsequent amplification and expression can be predicted [17, 18]. Therefore, we applied the evaluation of DV_200_ (RNA screen Tape Assays On Agilent 2200 Tape Station, Santa Cruz, CA, USA) which represents the percentage of RNA fragments above 200 nucleotides (a value of DV_200_ > 70% also being a good guarantee of RNA quality), to each sample of RNA. All these values and thresholds in terms of quality are strict standard requirements by most molecular biology platforms for an optimum and reliable molecular analysis.

#### Blood samples

Blood is sampled in EDTA and dry tubes (15 ml) and then treated as follows: centrifuge at 2200G for 15 min for plasma isolation (EDTA tubes), 2000xG for 10 min for serum isolation (dry tubes). Whole blood (EDTA tubes), serum and plasma are aliquoted and stored at − 80 °C until use. BACAP database can support ancillary projects with additional blood tubes that comply with the blood volume specified in the informed consent (35 ml). All blood aliquots are subsequently centralized in the Toulouse biorepository center for tumors (certified 96,900 - University Institute for Cancer Toulouse-Oncopole, France).

#### Saliva samples

Saliva is collected directly from the mouth using a 1 ml syringe, transferred in RNAprotect saliva Reagent® (Qiagen, Courtaboeuf, France) and stored at − 80 °C until use [19].

#### Pancreatic tissue samples

A specific tumor sample from surgery is kept specifically for the database.

#### Post- sampling process and quality control

Samples are prepared and frozen on the platforms and in the pathology departments of each center, which are all certified by recognized organizations, especially Inca.

##### Duration of the project

The project started in May, 2014 and has no specific end date. This database should be developed according to research advances and researcher request. A decision was initially made to include 1500 patients. This is the minimum size expected for this unique cohort in terms of design, data and samples. In addition, the database corresponds exactly to real life in terms of the medical care of PDAC patients.

##### Access to the database

Data and biological samples are collected to support large-scale research projects which aim to: i) validate in humans, hypotheses already proven in animals; ii) make correlations between biological and clinical data.

To access the database, projects should be submitted to the scientific committee by filling out the form available at: https://www.chu-toulouse.fr/-projet-bacap.

## Discussion

When this manuscript was submitted, 1140 patients were included in the BACAP cohort from 15 French centers. We recently performed an intermediary analysis of the first 703 inclusions (387 men - 55%, 316 women - 45%, median age, 70 years) for which all data are complete including patient follow-up. The clinical profile of the cohort appears to be representative of current practices in terms of diagnosis and treatment. The initial characteristics of tumors after pre-therapeutic investigations and curative surgery (when possible) were: resected in 17%, locally advanced in 32% and metastatic in 51% of the cases. The patients were treated as follows: chemotherapy 67% (including neo-adjuvant protocols in 10% of the cases), surgery most often followed by adjuvant chemotherapy 17%, best supportive care 16%. The median survival rates were 21, 15 and 9 months for resected, locally-advanced and metastatic PDAC patients respectively. From this preliminary extraction of clinical data we can conclude that our cohort corresponds to real life in terms of demography, treatments and prognosis.

For each patient we obtained all scheduled samples with very good harmonization of the procedures in each center in terms of sampling, transportation and conservation. In addition, all FNA samples allocated to nucleic acid extraction were centralized at a single center (Center of Toulouse) for harmonized extraction/purification, quantification and quality controls. Recently, a preliminary batch of samples was successfully tested with a good yield in terms of extraction and quantification.

From this preliminary analysis, we think the BACAP database is completely full in terms of epidemiological, clinical, radiological, pathological and follow-up data. In addition, to date, biological samples have been correctly collected and stored and will be of good quality for forthcoming research protocols (six have already started).

We hope that through the BACAP cohort, all on-going (and future) research programs will identify new molecular markers for PDAC diagnosis, prognosis, and maybe the predictive response to surgery and/or chemotherapy. This unique prospective biological clinical database also provides the opportunity to make correlations between the presence/expression of a wide panel of biomarkers (DNA, RNA, miRNA, proteins, etc.), epidemiological and social data, various clinical situations, different stages and tumor differentiation, treatments and survival (progression-free and overall survival). In addition, before any treatment the molecular analysis of the tumor itself can be correlated with those obtained simultaneously on saliva and circulating blood.

Recently, several consortiums performed genomic, transcriptomic, allelotypical and methylome analyses on PDAC tissues and the results were published as original contributions or introduced in a database [20–22]. However, most of these molecular data were generated from tissues of resected PDAC. The resected forms of PDAC represent 15 to 17% of the cases in real life [1] and the BACAP cohort provides the only opportunity to conduct wide-scale analyses on primary tumors from locally advanced and/or metastatic unresected tumors. To our knowledge, this kind of analysis does not exist and is one of the reasons why we established the BACAP cohort in order to collect molecular materials from EUS-FNA samples before any treatment. Thanks to the BACAP, the molecular profiles of resected or non-resected primary tumors, metastases and blood samples can be compared at the same time.

The BACAP cohort is unique in France and exceptional for pancreatic cancer considering the variety and amount of clinical data and biological samples prospectively collected. All items and samples are collected under quality control based on international standards including the harmonization of practices with database and biorepository centralization. The cohort is run by sound governance, which ensures the network’s sustainability. We meet the various criteria previously described by Demeure et al. concerning multi-institutional banking for pancreatic cancer: all regulatory issues have been successfully resolved, science is paramount, both academic and private centers make valuable contributions to the tissue/blood banking effort, all collaborations between pathologists, gastroenterologists, surgeons and researchers are effective [23]. We plan to broaden the scope of the BACAP to international teams as well as others European clinical biological bases for pancreatic diseases.

## Abbreviations

BACAP: Base clinico-biologique de l’adénocarcinome pancréatique (biological clinical pancreatic adenocarcinoma database)
EUS: Endoscopic ultrasound
FNAB: Fine needle aspiration biopsy
Inca: French national cancer institute
PDAC: Pancreatic ductal adenocarcinoma
